# Comparative effectiveness of glucagon-like peptide-1 agonists, dipeptidyl peptidase-4 inhibitors, and sulfonylureas on the risk of dementia in older individuals with type 2 diabetes in Sweden: an emulated trial study

**DOI:** 10.1016/j.eclinm.2024.102689

**Published:** 2024-06-20

**Authors:** Bowen Tang, Arvid Sjölander, Jonas W. Wastesson, Géric Maura, Pierre-Olivier Blotiere, Máté Szilcz, Jonathan K.L. Mak, Chenxi Qin, Michael Alvarsson, Dorota Religa, Kristina Johnell, Sara Hägg

**Affiliations:** a Department of Medical Epidemiology and Biostatistics, Karolinska Institutet, Stockholm, Sweden; b Department of Pharmacology and Pharmacy, The University of Hong Kong, Hong Kong SAR, China; c Division of Endocrinology and Diabetology, Department of Molecular Medicine and Surgery, Karolinska Institutet, Karolinska University Hospital, Stockholm, Sweden; d Division of Clinical Geriatrics, Department of Neurobiology, Care Sciences and Society (NVS), Karolinska Institutet, Stockholm, Sweden

**Keywords:** Antidiabetic drugs, Dementia, Emulated trial study

## Abstract

**Background:**

The comparative effectiveness of glucagon-like peptide-1 (GLP-1) agonists, dipeptidyl peptidase-4 (DPP-4) inhibitors, and sulfonylureas on the risk of dementia in older individuals with type 2 diabetes mellitus (T2DM) is unknown.

**Methods:**

We conducted a sequential trial emulation from 1st January 2010 to 30th June 2020 using data from Swedish national registers. Swedish residents who were aged 65 or older, had type 2 diabetes (T2DM), and initiated GLP-1 agonists, DPP-4 inhibitors, or sulfonylureas were followed for up to 10 years to assess the risk of dementia. Participants who had dementia, used the three drug classes, or had contraindications were excluded from enrollment. The characteristics between arms were balanced through the application of propensity scores estimated from predefined covariates. Intention-to-treat effects were analysed with all enrolled participants, while the per-protocol effects were analysed with participants who adhered to the assigned treatment.

**Findings:**

The pooled trial included 88,381 participants who received prescriptions for GLP-1 agonists (n = 12,351), DPP-4 inhibitors (n = 43,850), or sulfonylureas (n = 32,216) at baseline and were followed for an average of 4.3 years. A total of 4607 dementia cases developed during follow-up: 278 for the GLP-1 agonist initiators (incidence rate: 6.7 per 1000 person years), 1849 for DPP-4 inhibitor initiators (IR: 11.8), and 2480 for sulfonylurea initiators (IR: 13.7). In an intention-to-treat analysis, GLP-1 agonist initiation was associated with a reduced risk of dementia compared to sulfonylureas (hazard ratio: 0.69, 95% CI: 0.60–0.79, p < 0.0001) and DPP-4 inhibitors (HR: 0.77, 95% CI: 0.68–0.88, p < 0.0001), after adjusting for age, enrollment year, sex, socioeconomic factors, health conditions, and past medication uses. These findings were consistent in several sensitivity analyses, including a per-protocol analysis (HR for sulfonylureas: 0.41, 95% CI: 0.32–0.53, p < 0.0001; HR for DPP-4 inhibitors: 0.38, 95% CI: 0.30–0.49, p < 0.0001).

**Interpretation:**

Our research suggested that GLP-1 agonists were associated with a lower risk of dementia compared to sulfonylureas and DPP-4 inhibitors in older individuals with T2DM. Further clinical trials are needed to validate these findings.

**Funding:**

10.13039/501100004359Swedish Research Council, 10.13039/501100004047Karolinska Institutet, the 10.13039/100000049National Institute on Aging, the 10.13039/100000002National Institutes of Health, and 10.13039/501100004472Riksbankens Jubileumsfond.

**Research in context**

**Evidence before this study**

Glucagon-like peptide-1 (GLP-1) agonists and dipeptidyl peptidase-4 (DPP-4) inhibitors are incretin-based hypoglycemic drugs. We searched PubMed for publications in English using the terms “diabetes mellitus, type 2/drug therapy”, “cognition or cognition disorders”, and “randomised controlled trial (RCT)”. We identified four studies published before May 17th, 2024. Two secondary analyses of RCTs suggested that GLP-1 agonists could lower the risk of dementia in patients with type 2 diabetes mellitus (T2DM). Two other RCTs found no significant difference in cognitive outcomes when comparing DPP-4 inhibitors to a placebo or a sulfonylurea in patients with T2DM. The effectiveness of GLP-1 agonists and DPP-4 inhibitors in reducing dementia risk in patients with T2DM, compared to each other or sulfonylureas (the most common second-line antihyperglycemic class), remains to be clarified.

**Added value of this study**

This study emulates a three-arm RCT using data from Swedish national registers. It found that the use of GLP-1 agonists was associated with a 30% and 23% lower risk of dementia, respectively, compared with sulfonylureas and DPP-4 inhibitors.

**Implications of all the available evidence**

Our findings provide real-world evidence suggesting that GLP-1 agonists were associated with a lower risk of dementia in patients with T2DM, compared to DPP-4 inhibitors or sulfonylureas. These findings, which highlight the differences in effectiveness among these antihyperglycemic drugs on dementia risk, might inform the selection of antihyperglycemic therapy in patients with T2DM at risk for cognitive decline. However, there may be residual confounding from unknown or unmeasured factors for these findings. Clinical trials are needed to confirm our findings.

## Introduction

Type 2 diabetes mellitus (T2DM) is a chronic condition with high blood sugar levels caused by insulin resistance and/or inadequate insulin production.^1^ Individuals with T2DM have a 1.7-fold higher risk of developing dementia, leading to significant morbidity and mortality.^2^ Two newer classes of second-line hypoglycemic drugs, glucagon-like peptide-1 (GLP-1) agonists and dipeptidyl peptidase-4 (DPP-4) inhibitors, have gained increasing popularity among patients with T2DM.^3^ GLP-1 is a type of incretin released from the intestines after eating and rapidly broken down by the DPP-4 enzyme. GLP-1 helps regulate glucose metabolism by stimulating insulin secretion, reducing glucagon secretion, slowing gastric emptying, and promoting satiety.^4^ Two secondary analyses of randomized clinical trials (RCTs) found that GLP-1 agonists were associated with a lower risk of cognitive impairment and dementia compared to placebo in patients with T2DM.^5^^,^^6^ On the other hand, two other RCTs reported that DPP-4 inhibitors did not show a significant difference in cognitive outcomes compared to placebo or a sulfonylurea in patients with T2DM.^7^^,^^8^ Besides these RCTs, there is limited evidence from large-scale RCTs or real-world settings on the effectiveness of GLP-1 agonists and DPP-4 inhibitors compared to each other or sulfonylureas, the most commonly used second-line antihyperglycemic class, in dementia risk among patients with T2DM. Further research in real-world settings can help us better understand the comparative effectiveness of these hypoglycemic therapies and make more informed choices for patients with T2DM.

To address these needs, we analysed real-world data from the Swedish national registers to investigate the comparative effectiveness of these drug classes regarding dementia risk in older individuals with T2DM through a study design that emulates a clinical trial.

## Methods

### Study design, settings, and participants

We used the framework proposed by Hernán and Robins to simulate a three-arm RCT comparing the dementia risk among GLP-1 agonist, DPP-4 inhibitor, and sulfonylurea initiators.^9^ The trial emulation is specified in Table 1. We utilized data from a cohort that combined multiple Swedish national registers, including the National Patient Register (NPR), the National Prescribed Drug Register (NPDR), the Longitudinal Integrated Database for Health Insurance and Labor Market Studies (LISA), the National Education Register, and the National Register of Care and Social Services for the Elderly and Persons with Impairments. These registers were linked through a unique personal number. The National Patient Register provides comprehensive data on inpatient and specialized outpatient visits since 2001, with diagnoses classified according to the International Classification of Diseases, 10th version (ICD-10).^10^ The National Prescribed Drug Register contains information on all prescribed medications dispensed at pharmacies in Sweden since 2005, including the Anatomical Therapeutic Chemical (ATC) codes, dispensation date, and quantity dispensed.^11^ The cohort included Swedish residents aged 65 or older from 1st January 2004 to 31st December 2020, with over 3 million participants followed until December 2020. Retrospective data from 1st January 2004 or when participants turned 60 was also collected. The study follows to the Strengthening the Reporting of Observational Studies in Epidemiology (STROBE) Statement guidelines for reporting observational studies. The study was approved by the Regional Ethics Review Board in Stockholm (registration number: 2017/501-31). The requirement for informed consent was waived for this study based on Swedish national registers.

**Table 1:** Specification of target trial and its emulation using data from Swedish national registers.

Protocol component	Target trial specification	Target trial emulation
Eligibility criteria	• Adults aged ≥65 years old. • Have established type 2 diabetes. • Did not take sulfonylureas, DPP-4 inhibitors, or GLP-1 agonists in the past year. • No history of any types of dementia or cognitive impairment. • No history of chronic contraindications for GLP-1 agonists, DPP-4 inhibitors, or sulfonylureas, including inflammatory bowel disease, thyroid cancer, pancreatitis, pancreatic cancer, renal dysfunction, hepatic failure, intestinal bypass, and bariatric surgery. • Baseline is defined as the month in which all eligibility criteria are met.	• Adults aged ≥65 years old. • Have established type 2 diabetes, which was defined as having records of type 2 diabetes diagnosis (ICD-10: E11) or receiving a prescription of any non-insulin antidiabetic drugs (ATC: A10B), therefore excluding individuals with type 1 diabetes. • Without dispensation of sulfonylureas (ATC: A10BB), DPP-4 inhibitors (ATC: A10BH), or GLP-1 agonists (ATC: A10BJ) in the past year. • No records of any types of dementia (ICD-10: F00-03, G30, G31) or mild cognitive impairment (ICD-10: F06, R41) or receiving a prescription of anti-dementia drugs including donepezil (ATC: N06DA02), rivastigmine (N06DA03), galantamine (N06DA04), and memantine (N06DX01) in the past 5 years. • No records of contraindications for GLP-1 agonists, DPP-4 inhibitors, or sulfonylureas, including inflammatory bowel disease (ICD-10: K50, K51, K52), thyroid cancer (ICD-10: C73), pancreatitis (ICD-10: K85, K86.1), pancreatic cancer (ICD-10: C25), renal dysfunction (ICD-10: N17, N18.3, N18.4, N18.5, N18.9, N19, N00, N01, N03, N04, N05, I12.0, I13.1, I13.2, Y84.1, Z49, Z94.0, Z99.2), hepatic failure (K72), bariatric surgery (NOMESCO code for operations: JDFxx), and intestinal bypass (NOMESCO: JFDxx; ICD-10: Z98.0) in the past 5 years. • No immigration or emigration in the past 1 year to avoid unknown medication use outside Sweden. • Baseline is defined as the month in which all eligibility criteria are met.
Treatment strategies	Assignment to treatment of GLP-1 agonists, DPP-4 inhibitors, or sulfonylureas at baseline and remaining on the assigned drug without taking the other two drugs during follow-up. After treatment assignment, participants are allowed to receive advice or add glucose-lowering drugs, apart from the investigational drug classes, from investigators to manage their glucose concentrations according to the local guidelines.	Same as the target trial. Participants are allowed to receive advice or add hypoglycemics, apart from the investigational drug classes, from clinicians to manage their glucose concentrations according to the local guidelines during follow-up.
Treatment assignment	Individuals are randomly assigned to a strategy at baseline to balance the characteristics between arms.	Propensity scores are calculated based on predefined baseline covariates to balance the characteristics between treatment groups.
Outcomes	Time to dementia onset.	Same as the target trial.
Follow-up	Start at baseline and end at the month of dementia diagnosis, death, loss to follow-up, or end of follow-up, whichever occurs first.	Same as the target trial.
Causal contrast	Difference in risk of developing dementia over the follow-up period.	Same as the target trial.
Statistical analysis	• The intention-to-treat effect is estimated via a comparison of dementia risk among individuals who were assigned to each treatment strategy. • The per-protocol effect is estimated via a comparison of dementia risk among individuals who adhered to the treatment strategy during follow-up.	Same as the target trial.

The National Patient Registers provides comprehensive data on inpatient and specialized outpatient visits since 2001, which includes disease diagnoses, classified according to the International Classification of Diseases, 10th version (ICD-10), as well as the procedures performed during hospitalization, classified according to the NOMESCO Classification of Surgical Procedures (NOMESCO). The National Prescribed Drug Register provides information on all prescribed medications dispensed at pharmacies in Sweden since 2005, classified according to the Anatomical Therapeutic Chemical (ATC) codes. To address potential bias from different look-back periods in sequential trials, a fixed 5-year look-back period was used to assess disease histories in the inclusion criteria. GLP-1 agonists indicate glucagon-like peptide-1 agonists. DPP-4 inhibitors, dipeptidyl peptidase 4 inhibitors.

**Fig. 1:**
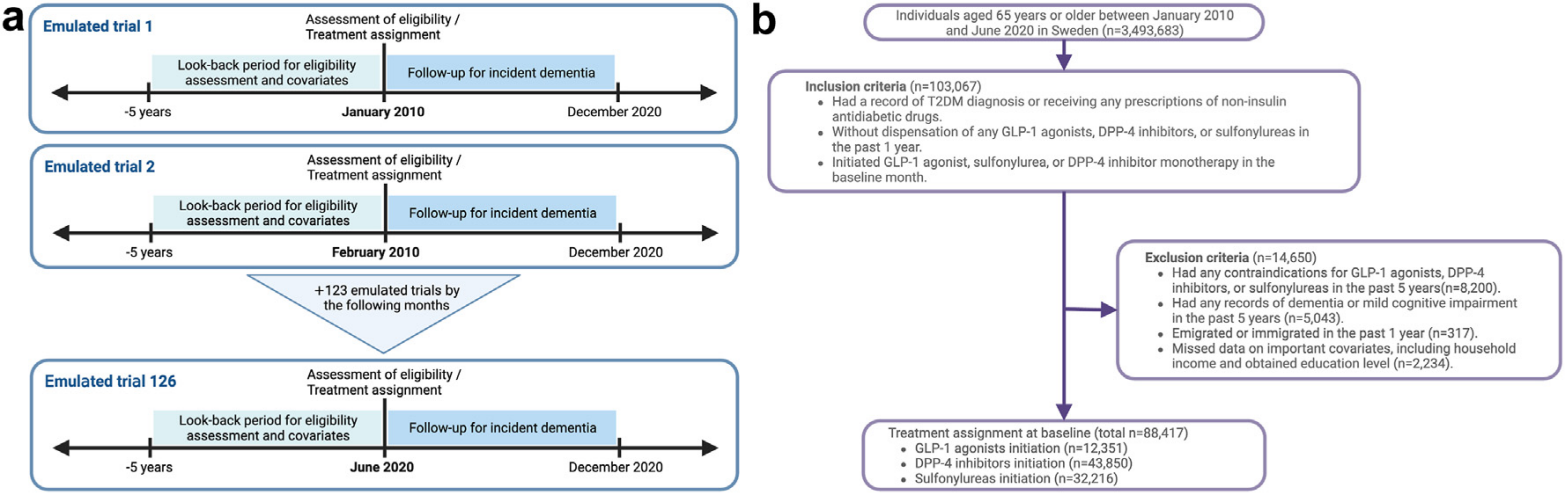
**Sequential trial emulation design and inclusion of participants**. a) Timeline of sequential trial emulation from January 2010 to June 2020. b) Inclusion and exclusion of participants for the pooled emulated trials. To maximize the sample size, we conducted a monthly sequential trial emulation from January 2010 to June 2020. Participants were treated as independent individuals each month. Those who met the eligibility criteria were included in the trial. To avoid bias resulting from varying look-back periods, we used a consistent look-back period of 5 years for the inclusion criteria in each trial. The numbers presented for the inclusion and exclusion of participants include repeated individuals, since participants meeting the eligibility criteria multiple times in the sequential trial emulation were enrolled in this study repeatedly. T2DM indicates type 2 diabetes mellitus. GLP-1 agonists, glucagon-like peptide-1 agonists. DPP-4 inhibitors, dipeptidyl peptidase 4 inhibitors.

To maximize the sample size for our study, we conducted a sequential trial emulation from 1st January 2010 to 30th June 2020. We initiated the emulation in 2010 to account for a three-year adjustment period for the use of GLP-1 agonists and DPP-4 inhibitors on the Swedish market, following their introduction in 2007. This also ensures a minimum look-back period of five years in our cohort to retrospectively identify disease histories and aligns with the launch of liraglutide in 2010, the most prescribed GLP-1 agonist until 2020 in Sweden. In the sequential trial emulation, participants were assessed monthly for eligibility. Those who met the criteria were included in the trial, with the first month as the “baseline”. Importantly, participants could meet the eligibility criteria in multiple months and be included multiple times e.g. if they stopped the treatments and restarted after one-year washout.^9^ Each month, participants from the cohort were included if they had a T2DM diagnosis or prescription for non-insulin hypoglycemic drugs before the month. Participants who had previously used the three studied medications were excluded, with a one-year washout period. Participants who had emigrated or immigrated within the past year were excluded to avoid unknown medication use outside Sweden. Participants with contraindications for the studied drug classes or records of dementia or mild cognitive impairment within the past 5 years were also excluded. See Table 1 for detailed eligibility criteria. To address potential bias from different look-back periods in sequential trials, a fixed 5-year look-back period was used to assess disease histories for each trial. This 5-year period has been validated in the Swedish registers as an effective means of identifying most disease histories.^12^ Additionally, a sensitivity analysis was performed to compare results using all available look-back periods starting from 2004. Fig. 1 depicts the sequential trial emulation and participant inclusion.

### Exposure and outcome

The study identified participants prescribed GLP-1 agonists (ATC codes for drugs: A10BJ), DPP-4 inhibitors (A10BH), or sulfonylureas (A10BB) at baseline. Incident dementia was identified through either diagnosis (ICD-10 codes for diseases: F00-03, G30, G31) or anti-dementia drug prescriptions (N06DA02, N06DA03, N06DA04, N06DX01). Similar to RCTs, participants can receive advice or additional non-investigational hypoglycemics from clinicians in accordance with local guidelines to manage their blood glucose levels during the follow-up period.^13^

### Covariates

Inverse-probability weighting (IPW) by the propensity scores was used to balance baseline characteristics between treatment groups. This involved assigning a weight to each participant based on their likelihood of receiving a dispensation for one of the three drugs, determined by their baseline characteristics.^14^ We identified potential confounders and prognostic factors for dementia in the registers.^15^ These factors included age, enrollment year, sex (identified from Swedish registers), and various health conditions for the past five years (hypertension, obesity, lifestyle-related conditions, heart diseases, hearing loss, sleep disorders, cerebrovascular diseases, cancers excluding localized skin cancers, neurological disorders, mental disorders, number of complications for T2DM, home care, chronic multimorbidity index, and the Hospital Frailty Risk Score). We also considered medication use for the past one year, including antihypertensive drugs, lipid-lowering drugs, cardiac therapy, psychotropic medications, antithrombotics, neuropharmaceuticals, metformin, insulin, sodium-glucose cotransporter-2 (SGLT2) inhibitors, thiazolidinediones, combinations of oral hypoglycemic drugs, other hypoglycemic drugs, and anticholinergic burden score. Socioeconomic factors such as household income (low, median, and high level based on three quantiles), education level (primary, secondary, and higher education), and civil status (having a partner or not), were taken into account. See the definition of covariates and classification codes in Supplementary Tables S1 and S2.

### Statistical methods

First, we pooled participants from all the trials and summarized their baseline characteristics. For the intention-to-treat analysis, the participants were followed until the first occurrence of dementia, death, loss to follow-up, or December 2020. We calculated overlap propensity scores for each participant using a multinomial logistic regression with predefined covariates.^14^^,^^16^ We then assessed the balance between groups by comparing the standardized mean difference (SMD) before and after weighting. We conducted a subgroup analysis focusing on initiators of liraglutide, which constituted 65% of GLP-1 agonist prescriptions in our sample. To account for a potential delay in registering dementia diagnoses, we conducted sensitivity analyses by censoring participants who received a dementia diagnosis within 1, 3, and 5 years from baseline.^17^ Additionally, we stratified the analysis by sex and age (65–74 years old and ≥75 years old), analysed participants with a T2DM diagnosis from the NPR within the past 5 years, and excluded participants diagnosed with any cerebrovascular diseases including stroke (G45, G46, I60, I61, I62, I63, I65, I66, I67, I68, I69). We extended the washout period to 2009 for an analysis that only considers the first initiation of the investigated treatments from 2010 to 2020 (no reuses of participants in sequential trial emulation). For per-protocol analysis, we considered both the treatment assignment and adherence. We estimated the duration of drug exposure using the dispensed quantity and prescribed daily dose in the NPDR. Participants were additionally censored after one month without refilling their assigned drugs or when they started taking the other two studied drug classes. Next, we estimated the stabilized propensity scores by considering the time-dependent probability of adherence to the protocol using a pooled logistic model.^18^ The scores were truncated at the 10th and 90th percentile before estimating the per-protocol effect.

We used the Kaplan–Meier method and Cox proportional hazard model to estimate both intention-to-treat and per-protocol effect after weighting with propensity scores. The proportional hazard assumption was tested using the Schoenfeld residuals. For absolute risk, we estimated the weighted risk difference per 1000 within a five-year period from baseline. We tested our trial emulation strategy with negative and positive control outcomes. Similar to dementia, chronic lower respiratory diseases (J40–47), hearing loss (H90, H91), and lens disorders (H25–28) are age-related conditions.^19^, ^20^, ^21^ However, there is no biological plausibility suggesting a relationship with the three studied medications, so they were used as negative control outcomes. Gastrointestinal reactions (R11, R12, R14, K59.0, K30) are considered adverse effects of GLP-1 agonists and were used as a positive control outcome. Since metformin is commonly used as monotherapy in early-stage T2DM, we analysed individuals who exclusively used metformin for blood glucose control in the year before baseline to ensure comparability of T2DM severity across the three groups (metformin-only users). Most GLP-1 agonists in Sweden until 2020 required injection, which doctors may consider when prescribing these drugs. An additional analysis included individuals who had used insulin in the year before baseline (insulin ever users). This analysis specifically compared GLP-1 agonists with DPP-4 inhibitors, as combining sulfonylureas with insulin is not recommended. To address bias in estimating standard errors caused by including the same individual multiple times in the sequential emulation, we used bootstrapping by resampling individuals 1000 times to generate confidence intervals with two-sided p < 0.05. See more details regarding statistical analysis in Supplementary Method.

### Patient and public involvement

There was no involvement of patients in setting the research question or in the design, conduct, or interpretation of the study. Since this study is based on anonymized nationwide register data, there are no plans to directly disseminate the results to study participants. However, a popular science press release will be issued on the Karolinska Institutet website.

### Role of the funding source

The funder of the study had no role in study design, data collection, data analysis, data interpretation, or writing of the report. The authors had full access to the data in the study and had final responsibility for the decision to submit for publication.

## Results

The pooled trial included 88,381 participants (81,369 unique individuals after accounting for repeated enrollment) who received prescriptions for GLP-1 agonists (n = 12,351), DPP-4 inhibitors (n = 43,850), or sulfonylureas (n = 32,216) at baseline and were followed for an average of 4.3 years. A total of 4607 dementia cases developed during follow-up: 278 for the GLP-1 agonist initiators (incidence rate (IR) = 6.7 per 1000 person years), 1849 for DPP-4 inhibitor initiators (IR = 11.8), and 2480 for sulfonylurea initiators (IR = 13.7) (Supplementary Table S3). The baseline characteristics are shown in Table 2. A significant imbalance (SMD >0.10) was observed for covariates including age, enrollment year, household income, education, marital status, hypertension, hyperlipidemia, obesity, sleep disorders, home care, chronic morbidity index, number of diabetic complications, and use of antihypertensives, lipid-lowering drugs, insulin, and SGLT2 inhibitors, which were balanced after IPW (SMD <0.10) (Table 2 and Supplementary Figure S1).

**Table 2:** Participant characteristics in GLP-1 agonist, DPP-4 inhibitor, and sulfonylurea groups at baseline.

	GLP-1 agonists	DPP-4 inhibitors	Sulfonylureas	Crude standardized mean difference			Weighted standardized mean difference		
				GLP-1 agonists vs. sulfonylureas	DPP-4 inhibitors vs. sulfonylureas	GLP-1 agonists vs. DPP-4 inhibitors	GLP-1 agonists vs. sulfonylureas	DPP-4 inhibitors vs. sulfonylureas	GLP-1 agonists vs. DPP-4 inhibitors
n	12,351	43,850	32,216						
Sex = Woman (%)	5138 (41.6)	19,731 (45.0)	14,588 (45.3)	0.074	0.006	0.069	0.027	0.017	0.010
Age at baseline (mean (SD))	71.62 (4.83)	74.78 (6.68)	74.21 (6.58)	0.449	0.087	0.543	0.004	0.040	0.037
Household income (%)				0.282	0.100	0.182	0.024	0.020	0.009
High	5155 (41.7)	14,547 (33.2)	9234 (28.7)						
Low	3343 (27.1)	14,412 (32.9)	11,544 (35.8)						
Median	3853 (31.2)	14,891 (34.0)	11,438 (35.5)						
Education (%)				0.243	0.080	0.165	0.016	0.031	0.016
Primary education	4065 (32.9)	17,894 (40.8)	14,324 (44.5)						
Secondary education	5692 (46.1)	18,066 (41.2)	12,783 (39.7)						
Tertiary education or higher	2594 (21.0)	7890 (18.0)	5109 (15.9)						
Civil status = Having a partner (%)	4009 (32.5)	10,388 (23.7)	5942 (18.4)	0.326	0.129	0.196	0.022	0.001	0.023
Enrollment year (%)				1.203	1.075	0.157	0.095	0.045	0.053
2010–2013	1844 (14.9)	6605 (15.1)	16,888 (52.4)						
2014–2017	4127 (33.4)	19,439 (44.3)	12,343 (38.3)						
2018–2020	6380 (51.7)	17,806 (40.6)	2985 (9.3)						
Cancers = Yes (%)	1150 (9.3)	4687 (10.7)	3227 (10.0)	0.024	0.022	0.046	0.004	0.001	0.004
Cerebrovascular diseases = Yes (%)	889 (7.2)	3640 (8.3)	2494 (7.7)	0.021	0.021	0.041	0.019	0.022	0.003
Hyperlipidemia = Yes (%)	2337 (18.9)	5886 (13.4)	3736 (11.6)	0.205	0.055	0.150	0.004	0.001	0.004
Hypertension = Yes (%)	6198 (50.2)	19,353 (44.1)	12,531 (38.9)	0.229	0.106	0.121	0.025	0.009	0.016
Heart diseases = Yes (%)	3934 (31.9)	12,704 (29.0)	8225 (25.5)	0.140	0.077	0.063	0.027	0.021	0.006
Neurological disorders = Yes (%)	343 (2.8)	1517 (3.5)	908 (2.8)	0.003	0.037	0.039	0.020	0.014	0.006
Hearing loss = Yes (%)	598 (4.8)	2056 (4.7)	1659 (5.1)	0.014	0.021	0.007	0.028	0.020	0.008
Mental disorders = Yes (%)	876 (7.1)	2829 (6.5)	1836 (5.7)	0.057	0.032	0.026	0.005	0.008	0.013
Obesity = Yes (%)	1641 (13.3)	1955 (4.5)	992 (3.1)	0.379	0.072	0.314	0.035	0.011	0.046
Sleep disorders = Yes (%)	1055 (8.5)	1823 (4.2)	1012 (3.1)	0.232	0.054	0.181	0.030	0.009	0.040
Lifestyle-related conditions = Yes (%)	428 (3.5)	1326 (3.0)	777 (2.4)	0.062	0.038	0.025	0.011	0.003	0.014
Antihypertensive drugs = Yes (%)	11,374 (92.1)	38,698 (88.3)	27,266 (84.6)	0.234	0.106	0.129	0.034	0.025	0.009
Lipid lowering drugs = Yes (%)	9402 (76.1)	30,157 (68.8)	19,853 (61.6)	0.317	0.150	0.165	0.008	0.023	0.014
Cardiac therapy = Yes (%)	2151 (17.4)	6926 (15.8)	5194 (16.1)	0.035	0.009	0.044	0.014	0.018	0.004
Psychotropic medications = Yes (%)	2157 (17.5)	6807 (15.5)	4453 (13.8)	0.100	0.048	0.052	0.028	0.003	0.024
Neuropharmaceuticals = Yes (%)	1044 (8.5)	2943 (6.7)	1756 (5.5)	0.118	0.053	0.066	0.017	0.001	0.016
Antithrombotics = Yes (%)	7190 (58.2)	23,785 (54.2)	16,585 (51.5)	0.136	0.055	0.080	0.036	0.031	0.006
Home cares = Yes (%)	1295 (10.5)	7441 (17.0)	4366 (13.6)	0.094	0.095	0.189	0.038	0.015	0.023
Chronic morbidity index (mean (SD))	4.34 (3.28)	3.82 (3.14)	3.38 (2.89)	0.314	0.147	0.164	0.045	0.018	0.026
HFRS (mean (SD))	1.80 (2.97)	1.80 (3.02)	1.47 (2.58)	0.117	0.117	0.001	0.045	0.032	0.012
ACB score (mean (SD))	6.11 (14.17)	6.32 (15.68)	5.80 (14.86)	0.021	0.034	0.014	0.051	0.028	0.024
No. of diabetic complications (mean (SD))	0.41 (0.78)	0.22 (0.55)	0.15 (0.43)	0.403	0.132	0.279	0.045	0.033	0.012
Metformin = Yes (%)	9472 (76.7)	35,626 (81.2)	26,389 (81.9)	0.129	0.017	0.112	0.040	0.040	0.001
Insulin = Yes (%)	7087 (57.4)	9253 (21.1)	2084 (6.5)	1.304	0.434	0.800	0.066	0.004	0.062
SGLT2 inhibitors = Yes (%)	1653 (13.4)	1807 (4.1)	337 (1.0)	0.491	0.195	0.332	0.020	0.016	0.035
Thiazolidinediones = Yes (%)	188 (1.5)	649 (1.5)	531 (1.6)	0.010	0.014	0.003	0.026	0.030	0.004
Combinations of oral antidiabetic drugs = Yes (%)	518 (4.2)	808 (1.8)	531 (1.6)	0.152	0.015	0.138	0.049	0.076	0.027
Other antidiabetic drugs = Yes (%)	691 (5.6)	2357 (5.4)	1035 (3.2)	0.116	0.107	0.010	0.042	0.004	0.046

GLP-1 agonists, glucagon-like peptide-1 agonists. DPP-4 inhibitors, dipeptidyl peptidase 4 inhibitors. HFRS, hospital frailty risk score. ACB score, anticholinergic burden score. SGLT2 inhibitors, sodium-glucose cotransporter-2 (SGLT2) inhibitors.

### Positive and negative controls

Supplementary Table S4 shows the results of negative and positive control analyses. For the positive control, GLP-1 agonists were associated with a 9% higher chance of adverse gastrointestinal reactions compared to sulfonylureas (Hazard ratio: 1.09, 95% CI: 0.99–1.21). For negative controls, we did not find a strong link for GLP-1 agonists and DPP-4 inhibitors with chronic lower respiratory disease, hearing loss, and lens disorders (HRs ranging from 0.97 to 1.07, p-values from 0.17 to 0.80).

### Intention-to-treat and per-protocol analysis

**Fig. 2:**
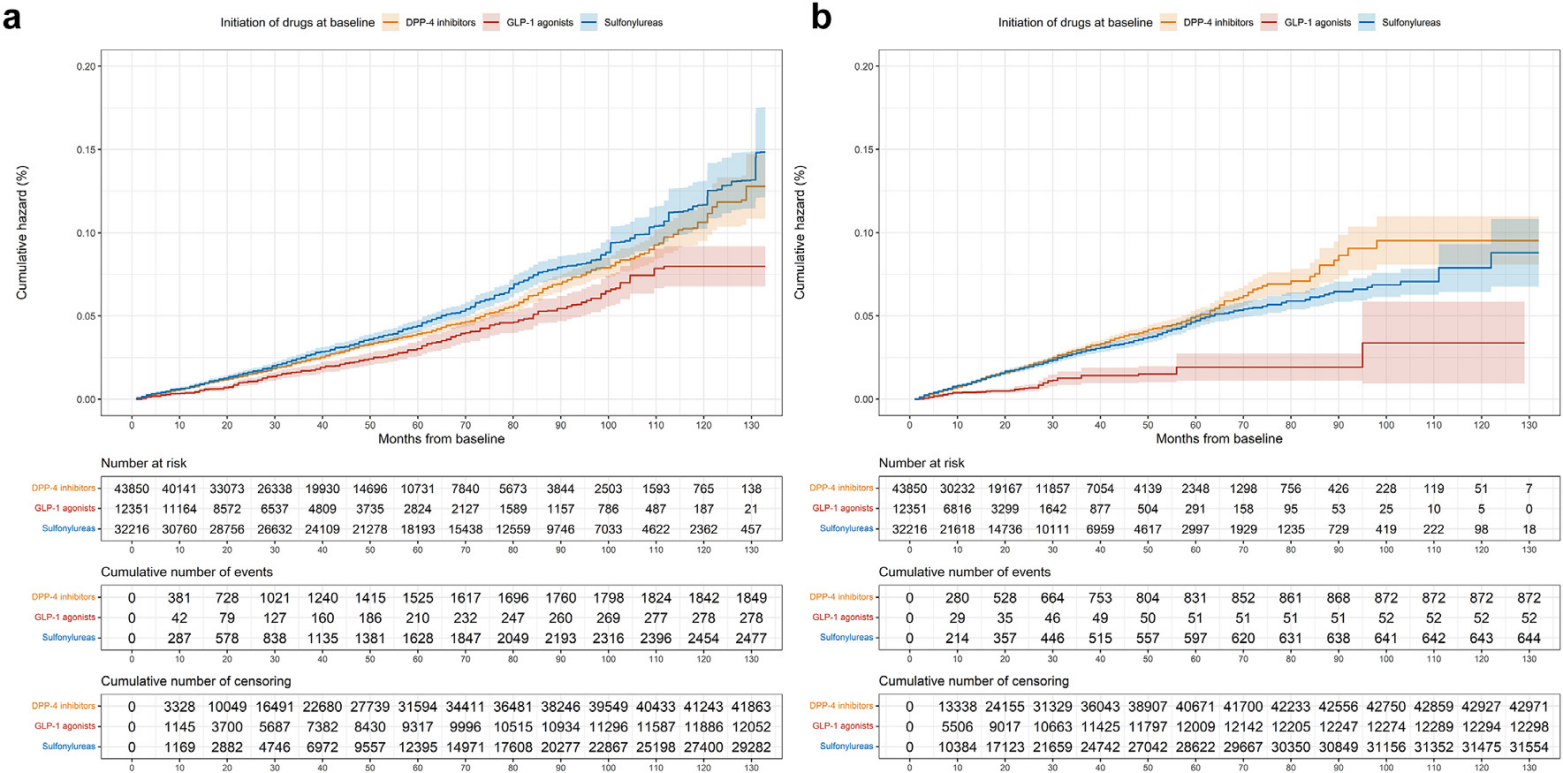
**Weighted cumulative hazard for incident dementia in GLP-1 agonist, DPP-4 inhibitor, and sulfonylurea groups**. a) Weighted cumulative hazard for dementia from intention-to-treat analysis. b) Weighted cumulative hazard for dementia from per-protocol analysis. The analysis included a total of 12,351 initiators of GLP-1 agonists, 43,850 initiators of DPP-4 inhibitors, and 32,216 initiators of sulfonylureas. For the per-protocol analysis, the adherence rates were 30% in the GLP-1 agonist group, 40% in the DPP-4 inhibitor group, and 16% in the sulfonylurea group. Intention-to-treat analysis was adjusted for the predefined baseline covariates, while per-protocol analysis further considered adherence to the assigned treatment with adjustment for the predefined baseline covariates and time-varying covariates along follow-up. GLP-1 agonists are glucagon-like peptide-1 agonists. DPP-4 inhibitors are dipeptidyl peptidase 4 inhibitors. Light-colored bands represent the 95% confidence interval for each treatment.

**Fig. 3:**
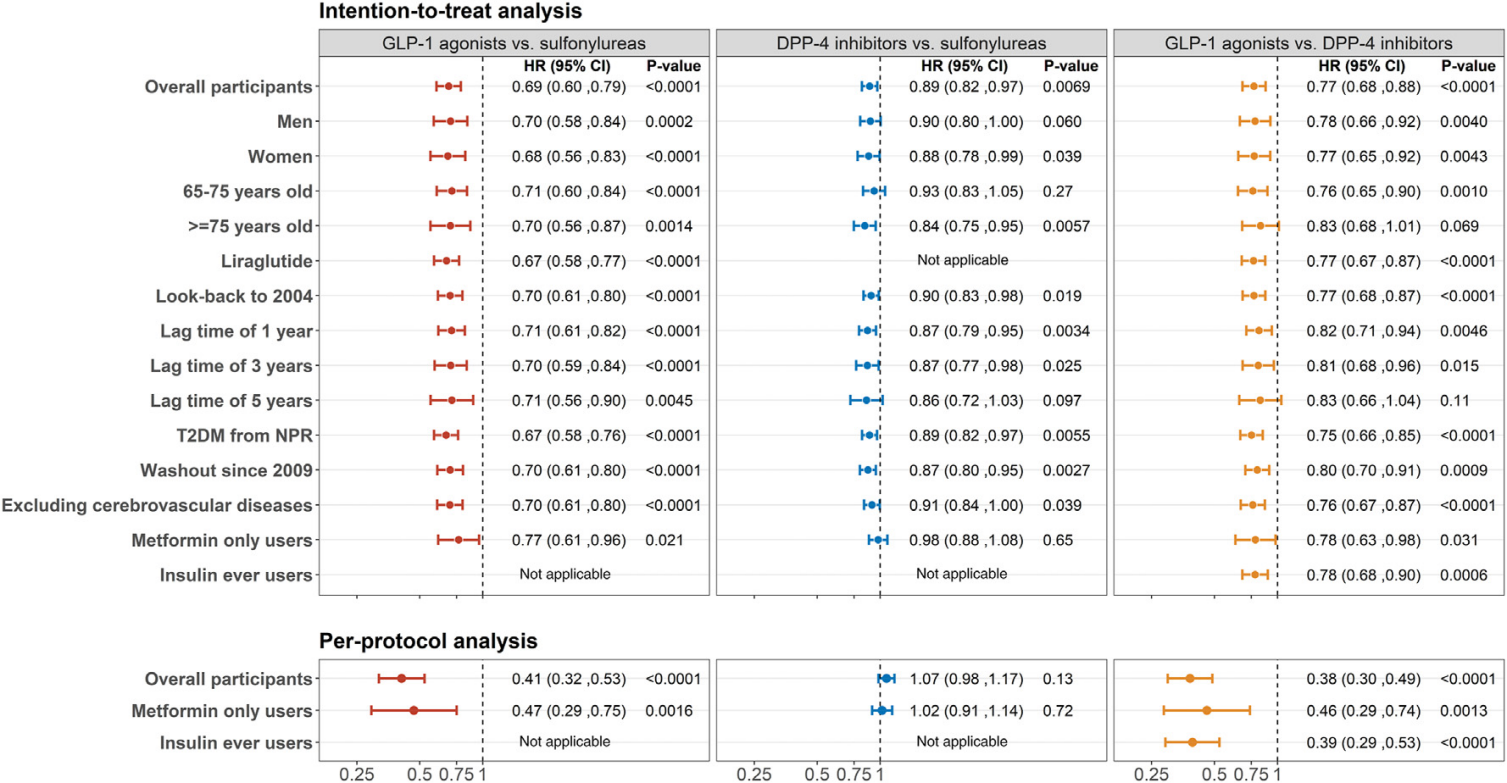
**Weighted hazard ratios for dementia for pairwise comparisons between GLP-1 agonist, DPP-4 inhibitor, and sulfonylurea groups for both intention-to-treat and per-protocol effects**. This figure illustrates weighted hazard ratios for dementia, as derived from both intention-to-treat (upper panel) and per-protocol analyses (lower panel). Pairwise comparisons were conducted using the latter drug as the reference. The intention-to-treat analysis of overall participants considered a five-year look-back period for eligibility assessment. Analyses of look-back to 2004 utilized an as-available look-back period to 2004 in our cohort. In the liraglutide analysis, only participants initiated on liraglutide were included in the GLP-1 agonist group, and the estimates for the comparison of liraglutide to DPP-4 inhibitors and sulfonylureas were provided. To account for possible register lag time in dementia diagnoses, analyses with lag times of 1, 3, and 5 years were conducted. Participants receiving a dementia diagnosis within 1, 3, or 5 years from baseline were censored accordingly. Additional analyses focused on specific subgroups by sex (men and women) and age (65–75 and ≥75 years old). The analysis concerning T2DM diagnosis in the past 5 years only included participants with T2DM records in the National Patient Register for the five years prior to baseline. The analysis with washout since 2009 extended the washout period to 2009 for an analysis that only considers the first initiation of the investigated treatments from 2010 to 2020 (no reuses of participants in sequential trial emulation). The analysis of excluding cerebrovascular diseases excluded the participants diagnosed with cerebrovascular diseases within the five years prior to baseline. The analysis of metformin only users only included the participants who exclusively received dispensation of metformin monotherapy in the preceding year to baseline, while the analysis of insulin users included the participants who ever used insulin within the year before baseline and had been performed only for comparison between GLP-1 agonists and DPP-4 inhibitors. All intention-to-treat analyses were adjusted for the predefined baseline covariates, while per-protocol analysis further considered adherence to the assigned treatment with adjustment for the predefined baseline covariates and time-varying covariates along follow-up. 'Not applicable' indicates the corresponding analysis was not feasible or necessary due to certain considerations. GLP-1 agonists refer to glucagon-like peptide-1 agonists. DPP-4 inhibitors are dipeptidyl peptidase 4 inhibitors.

The weighted cumulative hazard in each group is shown in Fig. 2a. GLP-1 agonists and DPP-4 inhibitors had a lower dementia risk compared to sulfonylurea initiators (HR for GLP-1 agonists: 0.69, 95% CI: 0.60–0.79; HR for DPP-4 inhibitors: 0.89, 95% CI: 0.82–0.97) (Figs. 2a and 3). GLP-1 agonists also had a lower risk compared to DPP-4 inhibitors (HR: 0.77, 95% CI: 0.68–0.88). Sensitivity analyses, considering a look-back period to 2004, registered lag time, liraglutide initiators, washout since 2009, excluding participants with cerebrovascular diseases, and T2DM diagnosis from NPR, yielded consistent results with the main analysis. Subgroup analyses based on sex and age also showed consistent results (Fig. 3).

In the per-protocol analysis, adherence rates were: 30% for GLP-1 agonists, 40% for DPP-4 inhibitors, and 16% for sulfonylureas. GLP-1 agonist initiators had a lower dementia risk compared to sulfonylureas (HR = 0.41, 95% CI: 0.32–0.53) and DPP-4 inhibitors (HR = 0.38, 95% CI: 0.30–0.49). No difference was observed between DPP-4 inhibitors and sulfonylureas (HR = 1.07, 95% CI: 0.98–1.17) (Figs. 2b and 3). At-risk time and event counts, both crude and after weighting, for the reported HRs are provided in Supplementary Table S3.

### Sensitivity analysis within users of metformin or insulin

In the sensitivity analysis of metformin only users, 3532 started using GLP-1 agonists, 26,581 started using DPP-4 inhibitors, and 23,475 started using sulfonylureas. Baseline characteristics are shown in Supplementary Table S5 and Supplementary Figure S2. Results for GLP-1 agonists in metformin only users were consistent with the main analysis, suggesting protective effects in both intention-to-treat and per-protocol analysis compared with either sulfonylureas or DPP-4 inhibitors (HRs ranging from 0.46 to 0.78). However, the estimates for DPP-4 inhibitors compared with sulfonylureas diminished to null in either intention-to-treat (HR = 0.98, 95% CI = 0.88–1.08) or per-protocol analysis (HR = 1.02, 95% CI = 0.91–1.14) (Fig. 3). In the sensitivity analysis of insulin ever users, 7087 started using GLP-1 agonists and 9253 started using DPP-4 inhibitors. Baseline characteristics are provided in Supplementary Table S6 and Supplementary Figure S3. Results showed a lower risk for GLP-1 agonists compared to DPP-4 inhibitors in both intention-to-treat (HR: 0.75, 95% CI: 0.55–1.02) and per-protocol analysis (HR: 0.41, 95% CI: 0.24–0.71) (Fig. 3). No violation of the proportional hazard assumption was detected for the reported HRs (all p-values for Schoenfeld residual test >0.05).

The absolute risk difference among overall participants, metformin-only users, and insulin ever users can be found in Supplementary Table S7. These results align with the HR estimates, showing a lower dementia risk for GLP-1 agonists compared to sulfonylureas or DPP-4 inhibitors (5-year risk difference: −13.81 to −4.37 per 1000).

## Discussion

This study was the first to compare the effects of GLP-1 agonists, DPP-4 inhibitors, and sulfonylureas on the risk of dementia in older patients with T2DM, using a large sample from nationwide registers and an emulated trial design. Our findings revealed that GLP-1 agonists were associated with a lower risk of dementia in patients with T2DM, compared to DPP-4 inhibitors or sulfonylureas. Subgroup and sensitivity analyses further supported these findings.

There is limited evidence regarding the effectiveness of GLP-1 agonists compared to DPP-4 inhibitors or sulfonylureas regarding dementia risk in patients with T2DM. Two secondary analyses were conducted based on the samples from previous RCTs that examined the cardiovascular effects of GLP-1 agonists in patients with T2DM, with approximately 10,000 participants randomly assigned to either GLP-1 agonists or placebo.^5^^,^^6^ Similar to our emulated trial, the participants in these RCTs were allowed to take additional non-investigational drugs to control blood glucose levels after treatment assignment, ensuring that the estimated drug effects were not influenced by poor blood glucose management in any group. The studies showed that GLP-1 agonists were associated with a 54% lower risk of dementia and a 14% lower risk of cognitive impairment compared to placebo. In real-world settings, a study conducted using Danish registers employed a nested case–control design to assess the effects of GLP-1 agonists by comparing prevalent users and non-users in patients with T2DM and dementia and their matched controls.^22^ The study showed that exposure to GLP-1 agonists was associated with a reduced risk of dementia (HR: 0.58, 95% CI: 0.50–0.67). However, this retrospective design differs from our emulated trial, in which we identified new users in the same month, followed them until the onset of dementia, and compared them with the users of other two second-line hypoglycemic drug classes. By incorporating this prospective new-user design with active comparators, our study is better positioned to provide more reliable estimates regarding the effects of GLP-1 agonists on dementia relative to DPP-4 inhibitors and sulfonylureas.^23^^,^^24^

Our study also noted a slightly reduced risk for DPP-4 inhibitors compared to sulfonylureas (HR = 0.89, 95% CI = 0.82–0.97). However, when restricting to metformin only users, the effect was not sustained (HR = 1.02, 95% CI = 0.91–1.14), which indicates a possible confounding effect of T2DM severity on the estimated effects of DPP-4 inhibitors. In addition, an RCT found no significant difference in cognitive outcomes between patients with T2DM who were assigned to a DPP-4 inhibitor or sulfonylurea.^7^ Thus, it is challenging to draw a definitive conclusion about the effectiveness of DPP-4 inhibitors on dementia from our results and the existing trial evidence. Yet, when comparing GLP-1 agonists to DPP-4 inhibitors, both of which are incretin-based drugs introduced to Sweden in the same year and recommended as second-line hypoglycemics, we consistently found that GLP-1 agonists had superior effects to DPP-4 inhibitors in various analyses. DPP-4 inhibitors suppress the DPP-4 enzyme to prolong the half-life of endogenous GLP-1. This mechanism is, however, less effective compared to GLP-1 agonists, which directly activate GLP-1 receptors and provide supraphysiological responses.^25^ Besides, numerous RCTs have acknowledged the cardiovascular benefits and weight loss properties of GLP-1 agonists in patients with T2DM, which were not evident for DPP-4 inhibitors.^25^ Notably, most GLP-1 agonists can cross the blood–brain barrier and activate GLP-1 receptors in the brain.^26^ This activation helps regulate various processes that are affected in neurodegenerative diseases, such as neuroinflammation, oxidative stress, apoptosis, and dysfunction of brain glucose metabolism.^27^ These facts collectively highlight the distinct effects of GLP-1 agonists and reinforce our findings that GLP-1 agonists may indeed provide more substantial protective properties against dementia than DPP-4 inhibitors.

The strength of our study lies in the use of nationwide real-world data, the emulated trial design, and the long follow-up period of up to ten years to assess dementia risk. We followed the target trial emulation framework, defining the start of follow-up as the point when eligibility criteria were met and treatment was assigned, thereby minimizing time-related biases.^28^ We considered important factors such as age, sex, socioeconomic status, health conditions, and medication uses to ensure a balanced comparison between groups. In addition to intention-to-treat effect, we also estimated the per-protocol effect, which could offer a more accurate reflection of the drug's effects.^18^ To validate our trial design, we analysed positive and negative control outcomes. We also performed sensitivity and subgroup analyses to ensure reliable findings in different scenarios. Specifically, we examined metformin only and insulin ever users to address potential bias due to imbalances in T2DM severity and GLP-1 agonist injection requirement.

Our study has some limitations. First, despite our efforts to balance participant characteristics by including numerous factors from the registers, there may still be residual confounding due to unmeasured factors or imprecise measurements. Specifically, because GLP-1 agonists are recommended for patients with T2DM and cardiovascular disorders, a higher cardiovascular risk in the GLP-1 agonist group may remain even after adjusting for important cardiometabolic factors in our study. Since cardiovascular disorders are considered risk factors for dementia, such residual confounding would have skewed the risk estimates towards the null or higher,^29^ which is contrary to the lower risk observed in our study. Second, our cohort does not include lifelong data for the participants, which hinders our ability to assess T2DM duration as an indicator of T2DM severity to be adjusted for in our analysis. However, we used the number of diabetic complications as a substitute in our analysis. Additionally, we conducted a sensitivity analysis in the metformin only users to ensure comparability of T2DM severity across the groups. Moreover, our study utilized dementia diagnoses provided by the NPR. A study has reported a high level of specificity and positive predictive value, but moderate sensitivity in identifying dementia in the NPR.^17^ To address the issue of underdiagnosis of dementia in the NPR, we supplemented the dementia identification by incorporating the use of anti-dementia medications in the NPDR, as the NPDR provides greater accuracy and wider coverage by automatically collecting data on all medications dispensed by pharmacies in Sweden. However, there was still a high misclassification rate between the diagnosis for different dementia subtypes in the NPR, which, in turn, restricted our ability to thoroughly investigate specific dementia subtypes.^17^ Furthermore, this study estimated treatment effects by comparing three classes of antidiabetic drugs, which may not reflect the absolute effects of each drug. For instance, hypoglycemia is a common side effect of sulfonylureas, but is rare with GLP-1 agonists and DPP-4 inhibitors, which might mediate the relative risk difference when compared to sulfonylureas.^30^^,^^31^ However, GLP-1 agonists also demonstrated a lower dementia risk compared to DPP-4 inhibitors, suggesting no or minimal influence from hypoglycemia. Nevertheless, additional trials comparing these drugs with placebos are required to evaluate their absolute impact on dementia. Besides, we focused on GLP-1 agonists, DPP-4 inhibitors, and sulfonylureas in this study to maintain comparability. These are all second-line antidiabetic drugs, differing from metformin, which is the only first-line and commonly used for early-stage T2DM, and insulin, typically used at late-stage T2DM. SGLT2 inhibitors, the newest second-line antidiabetic drugs, were introduced to Sweden in 2012, much later than 2008 when GLP-1 agonists and DPP-4 inhibitors were launched. Finally, our study was conducted in Swedish residents aged 65 years or older with T2DM, which may limit the generalizability of our findings.

In this emulated trial involving 88,381 older patients with T2DM, we found real-word evidence suggesting that the use of GLP-1 agonists was associated with a reduced risk of developing dementia compared with DPP-4 inhibitors and sulfonylureas. Our findings suggest the potential effectiveness of GLP-1 agonists in preventing dementia in patients with T2DM. Indeed, several pilot RCTs conducted in cognitively impaired individuals without T2DM have found that GLP-1 agonists enhance hippocampal connections, cerebral glucose metabolism, and hippocampal activation.^27^ Herein, we hypothesized that the beneficial effects of GLP-1 agonists may extend beyond their specific effects on patients with T2DM. However, it is important to note that further clinical trials are necessary to validate our findings and assess their applicability to other populations.

## Contributors

BT and SH initiated the study. They had access to and verified the underlying study data. They are responsible for ensuring the integrity and accuracy of the data analysis. They affirm that the manuscript provides an honest, accurate, and transparent account of the reported study and that no important aspects of the study have been omitted. BT performed the statistical analysis and drafted the manuscript. All authors contributed to the study's design, analysis, and interpretation of data, as well as the critical revision of the manuscript. SH supervised the study and is the guarantor. The corresponding author confirms that all listed authors meet the criteria for authorship and that no other authors meeting the criteria have been excluded.

## Data sharing statement

The data supporting the findings of this study contain sensitive information. Due to these restrictions, and under the license used for this study, the data are not publicly available. In accordance with Swedish laws, the authors are not allowed to share the datasets with third parties. However, with appropriate ethical approval, individuals can apply for data from Statistics Sweden (http://www.scb.se/en/) and the National Board of Health and Welfare (https://www.socialstyrelsen.se/en/).

## Declaration of interests

All authors have provided signed conflict of interest forms and declared no competing interests.

## Appendix A Supplementary data

Supplements

Translated abstract in Swedish
