# Supplementary material for: Comparative effectiveness of glucagon-like peptide-1 agonists, dipeptidyl peptidase-4 inhibitors, and sulfonylureas on the risk of dementia in older individuals with type 2 diabetes in Sweden: an emulated trial study
Source: eClinicalMedicine. 2024 Jun 20;73:102689. doi: 10.1016/j.eclinm.2024.102689 (PMC11490655; doi:10.1016/j.eclinm.2024.102689)
Supplement: Supplements [file mmc1.pdf]

Supplementary Table 1. Classification of health-related conditions included as covariates in this study. All these health-related conditions were defined based on the data from National Patient Register within 5 years before baseline for analyses in the study, excluding the one with look-back period to 2004.

<table>
<tr><th>Health-related conditions</th><th colspan="2">ICD-10 codes</th><th>Variable type</th></tr>
<tr><td>Hypertension</td><td colspan="2">I10-15</td><td rowspan="23">Binary, defined based on the presence of the ICD-10 codes for the corresponding category in the National Patient Register.</td></tr>
<tr><td>Disorders of lipoprotein metabolism</td><td colspan="2">E78</td></tr>
<tr><td>Obesity</td><td colspan="2">E66, E65</td></tr>
<tr><td rowspan="3">Lifestyle-related conditions</td><td>Lifestyle-related conditions</td><td>Z72</td></tr>
<tr><td>Smoking abuse</td><td>F17</td></tr>
<tr><td>Alcoholism</td><td>F10, Y90, Y91</td></tr>
<tr><td rowspan="4">Heart diseases</td><td>Atrial fibrillation</td><td>I48</td></tr>
<tr><td>Ischemic heart disease</td><td>I20, I21, I22, I23, I24, I25</td></tr>
<tr><td>Heart failure</td><td>I50</td></tr>
<tr><td>Aortic aneurysm and dissection</td><td>I71</td></tr>
<tr><td>Hearing loss</td><td colspan="2">H90, H91</td></tr>
<tr><td>Number of complications for type 2 diabetes</td><td colspan="2">E11.0-11.8</td></tr>
<tr><td>Sleep disorders</td><td colspan="2">F51, G47</td></tr>
<tr><td>Cerebrovascular diseases</td><td colspan="2">G45, G46, I60, I61, I62, I63, I65, I66, I67, I68, I69</td></tr>
<tr><td>Cancers excluding localized skin cancers</td><td colspan="2">Cxx except for C44.xx</td></tr>
<tr><td rowspan="7">Neurological disorders</td><td>Multiple sclerosis</td><td>G35</td></tr>
<tr><td>Huntington's disease</td><td>G10</td></tr>
<tr><td>Intracranial injury</td><td>S06</td></tr>
<tr><td>Parkinson’s disease</td><td>G20</td></tr>
<tr><td>Brain tumor</td><td>D32, D33, D42, D43, C71</td></tr>
<tr><td>Epilepsy</td><td>G40</td></tr>
<tr><td>Migraine</td><td>G43</td></tr>
<tr><td>Mental disorders</td><td colspan="2">F04-09, F10-19, F20-29, F30-39, F40-48, F50-59, F60-69, F80-89, F90-98, F99</td></tr>
<tr><td>Number of diabetic complications</td><td colspan="2">E11.0-11.8</td><td>Continuous variable, defined as the number of each unique ICD-10 code for diabetic complications presented in the National Prescribed Drug Register.</td></tr>
<tr><td>Chronic morbidity index</td><td colspan="2">Based on the codes from Amaia Calderón-Larrañaga et al.’s study[1]. See details there.</td><td>Continuous variable as scores.</td></tr>
<tr><td>The Hospital Frailty Index</td><td colspan="2">Based on the codes from Gilbert T et al's study[2]. See details there.</td><td>Continuous variable as scores.</td></tr>
</table>

1. Calderón-Larrañaga A, et al. *J Gerontol A Biol Sci Med Sci*. 2017 Oct 1;72(10):1417-1423.
2. Gilbert T, et al. *Lancet*. 2018 May 5;391(10132):1775-1782.

Supplementary Table 2. Classification of the medication use included as covariates in this study. The medication uses were identified within one year before baseline.

Drug classes		ATC code	Variable type
Antihypertensive drugs	Antihypertensives	C02	Binary, defined based on the presence of the ATC codes for the corresponding category in the National Prescribed Drug Register.
	Diuretics	C03	
	Peripheral vasodilators	C04	
	Vasoprotectives	C05	
	Beta blocking agents	C07	
	Agents acting on the renin-angiotensin system	C09	
	Calcium channel blockers	C08	
Lipid lowering drugs	Statins	C10AA	
	Other lipid lowering drugs	C10AB, C10AC, C10AD, C10AX	
Psychotropic medications	Antidepressants	N06A	
	Antipsychotics	N05A	
Cardiac therapy		C01	
Antithrombotics	Antiplatelet agents	B01AC06	
		B01AC04	
		B01AC24	
		B01AC22	
	Anticoagulants	B01AA03	
		B01AB01	
		B01AB05	
		B01AE07	
		B01AF01	
		B01AF02	
		B01AF03	
	Thrombolytics for acute ischemic stroke	B01AD02	
		B01AD04	
		B01AD11	
Neuropharmaceuticals	Anti-Parkinson	N04	
	Antiepileptics	N03A	
	Anti-migraine drugs	N02C	
	Drugs for ALS	N07XX02, N07XX59	
	Drugs for Huntington's disease	N07XX06, N07XX10	
Antidiabetic drugs	Metformin	A10BA02	
	Insulin	A10A	
	SGLT2 inhibitors	A10BK	
	Thiazolidinediones	A10BG	
	Combinations of oral antidiabetic drugs	A10BD	
	Other antidiabetic drugs	A10BC (sulfonamides), A10BF (alpha glucosidase inhibitors), A10BX (other blood glucose lowering drugs, excl. insulins)	
Anticholinergic burden score		Based on scale with ATC codes from Tan ECK, et al's study[1]. See details there.	Continuous variable as scores

1. Tan ECK, et al. J Alzheimers Dis. 2018;65(2):589-596.

Supplementary Table 3. Risk-time, event counts, incidence rate and hazard ratios for dementia for pairwise comparisons between GLP-1 agonist, DPP-4 inhibitor, and sulfonylurea groups for both intention-to-treat and per-protocol effects. GLP-1 agonists refer to glucagon-like peptide-1 agonists. DPP-4 inhibitors are dipeptidyl peptidase 4 inhibitors. Pairwise comparisons were conducted using the latter drug as the reference. The intention-to-treat analysis of overall participants considered a five-year look-back period for eligibility assessment. Analyses of look-back to 2004 utilized an as-available look-back period to 2004 in our cohort. In the liraglutide analysis, only participants initiated on liraglutide were included in the GLP-1 agonist group, and the estimates for the comparison of liraglutide to DPP-4 inhibitors and sulfonylureas were provided. To account for possible register lag time in dementia diagnoses, analyses with lag times of 1, 3, and 5 years were conducted. Participants receiving a dementia diagnosis within 1, 3, or 5 years from baseline were censored accordingly. Additional analyses focused on specific subgroups by sex (men and women) and age (65-75 and >=75 years old). The analysis with washout since 2009 extended the washout period to 2009 for an analysis that only considers the first initiation of the investigated treatments from 2010 to 2020 (no reuses of participants in sequential trial emulation). The analysis concerning T2DM diagnosis in the past 5 years only included participants with T2DM records in the National Patient Register for the five years prior to baseline. The analysis of metformin only users only included the participants who exclusively received dispensation of metformin monotherapy in the preceding year to baseline, while the analysis of insulin users included the participants who ever used insulin within the year before baseline and had been performed only for comparison between GLP-1 agonists and DPP-4 inhibitors. All intention-to-treat analyses were adjusted for the predefined baseline covariates, while per-protocol analysis further considered adherence to the assigned treatment with adjustment for the predefined baseline covariates and time-varying covariates along follow-up. 'Not applicable' indicates the corresponding analysis was not feasible or necessary due to certain considerations.

	Crude									Weighted									Weighted HR (95%CI)		
	GLP-1 agonists			DPP-4 inhibitors			Sulfonylureas			GLP-1 agonists			DPP-4 inhibitors			Sulfonylureas			GLP-1 agonists vs. sulfonylureas	DPP-4 inhibitors vs. sulfonylureas	GLP-1 agonists vs. DPP-4 inhibitors
	Incident dementia	Person years	Incidence rate per 1000	Incident dementia	Person years	Incidence rate per 10000	Incident dementia	Person years	Incidence rate per 1000	Incident dementia	Person years	Incidence rate per 1000	Incident dementia	Person years	Incidence rate per 10000	Incident dementia	Person years	Incidence rate per 1000			
**Intention to treat**																					
Overall participants	278.0	41672.2	6.7	1849.0	157301.1	11.8	2480.0	181569.5	13.7	26.9	3987.9	6.7	33.6	3946.8	8.5	37.5	3962.0	9.5	0.69 (0.60 ,0.79)	0.89 (0.82 ,0.97)	0.77 (0.68 ,0.88)
Men	159.0	23710.1	6.7	922.0	86686.3	10.6	1251.0	98912.1	12.6	14.5	2245.8	6.5	17.8	2207.8	8.1	20.0	2231.2	9.0	0.70 (0.58 ,0.84)	0.90 (0.80 ,1.00)	0.78 (0.66 ,0.92)
Women	119.0	17962.1	6.6	927.0	70614.8	13.1	1229.0	82657.4	14.9	12.3	1729.5	7.1	15.6	1736.6	9.0	17.3	1720.1	10.0	0.68 (0.56 ,0.83)	0.88 (0.78 ,0.99)	0.77 (0.65 ,0.92)
65-75 years old	164.0	34474.5	4.8	620.0	101312.0	6.1	864.0	119331.6	7.2	15.8	3349.3	4.7	19.4	3281.9	5.9	21.1	3337.4	6.3	0.71 (0.60 ,0.84)	0.93 (0.83 ,1.05)	0.76 (0.65 ,0.90)
>=75 years old	114.0	7197.7	15.8	1229.0	55989.1	22.0	1616.0	62237.9	26.0	10.7	676.4	15.8	12.7	674.2	18.8	14.8	666.3	22.3	0.70 (0.56 ,0.87)	0.84 (0.75 ,0.95)	0.83 (0.68 ,1.01)
Liraglutide	213.0	32691.4	6.5	1849.0	157301.1	11.8	2480.0	181569.5	13.7	22.6	3429.3	6.6	29.5	3467.4	8.5	32.5	3394.4	9.6	0.67 (0.58 ,0.77)	Not applicable	0.77 (0.67 ,0.87)
Look-back to 2004	267.0	40598.2	6.6	1782.0	153710.1	11.6	2417.0	179255.6	13.5	26.1	3913.7	6.7	32.6	3879.7	8.4	35.8	3896.5	9.2	0.70 (0.61 ,0.80)	0.90 (0.83 ,0.98)	0.77 (0.68 ,0.87)
Lag time of 1 year	233.0	41672.2	5.6	1393.0	157301.1	8.9	2147.0	181569.5	11.8	23.6	3987.9	5.9	27.6	3946.8	7.0	31.3	3962.0	7.9	0.71 (0.61 ,0.82)	0.87 (0.79 ,0.95)	0.82 (0.71 ,0.94)
Lag time of 3 years	131.0	41672.2	3.1	687.0	157301.1	4.4	1455.0	181569.5	8.0	14.4	3987.9	3.6	15.8	3946.8	4.0	17.6	3962.0	4.4	0.70 (0.59 ,0.84)	0.87 (0.77 ,0.98)	0.81 (0.68 ,0.96)
lag time of 5 years	68.0	41672.2	1.6	324.0	157301.1	2.1	852.0	181569.5	4.7	8.6	3987.9	2.1	8.3	3946.8	2.1	8.8	3962.0	2.2	0.71 (0.56 ,0.90)	0.86 (0.72 ,1.03)	0.83 (0.66 ,1.04)
Washout since 2009	267.0	37094.2	7.2	1747.0	132136.6	13.2	2369.0	158154.8	15.0	25.2	3444.3	7.3	32.4	3392.8	9.5	36.2	3402.2	10.6	0.67 (0.58 ,0.76)	0.89 (0.82 ,0.97)	0.75 (0.66 ,0.85)
T2DM from NPR	199.0	30561.8	6.5	1509.0	132846.1	11.4	1990.0	155107.3	12.8	21.2	3153.8	6.7	25.8	3152.3	8.2	29.6	3189.6	9.3	0.70 (0.61 ,0.80)	0.87 (0.80 ,0.95)	0.80 (0.70 ,0.91)
Metformin only users	60.0	11645.7	5.2	982.0	96888.8	10.1	1645.0	135567.8	12.1	10.1	1876.3	5.4	13.0	1936.7	6.7	13.8	2011.5	6.8	0.77 (0.61 ,0.96)	0.98 (0.88 ,1.08)	0.78 (0.63 ,0.98)
Insulin ever users	196.0	303565.1	0.6	513.0	403912.0	1.3	Not applicable			118.6	159917.0	0.7	146.8	156738.2	0.9	Not applicable			Not applicable	Not applicable	0.78 (0.68 ,0.90)
**Per-protocol**																					
Overall participants	52.0	14798.7	3.5	872.0	77351.3	11.3	644.0	64257.5	10.0	45.2	11223.4	4.0	786.6	75915.1	10.4	446.4	48014.2	9.3	0.41 (0.32 ,0.53)	1.07 (0.98 ,1.17)	0.38 (0.30 ,0.49)
Metformin only users	14.0	4446.1	3.1	451.0	49379.8	9.1	420.0	49540.6	8.5	16.4	4216.0	3.9	420.5	50059.2	8.4	327.3	41087.2	8.0	0.47 (0.29 ,0.75)	1.02 (0.91 ,1.14)	0.46 (0.29 ,0.74)
Insulin ever users	35.0	8468.4	4.1	226.0	15007.1	15.1	Not applicable			39.8	7905.8	5.0	186.6	14541.5	12.8	Not applicable			Not applicable	Not applicable	0.39 (0.29 ,0.53)

Supplementary Table 4. Weighted hazard ratios from the analysis of positive and negative outcome controls for the intention-to-treat effects in the overall participants.

Analysis	GLP-1 agonists vs. sulfonylureas	P-value	DPP-4 inhibitors vs. sulfonylureas	P-value
**Positive control**				
Adverse gastrointestinal reactions	1.09 (0.99,1.21)	0.07	1.02 (0.95,1.10)	0.50
**Negative control**				
Chronic lower respiratory disease	1.07 (0.97,1.18)	0.17	1.02 (0.95,1.10)	0.61
Hearing loss	1.02 (0.90,1.16)	0.73	0.97 (0.88,1.06)	0.46
Disorders of lens	1.01 (0.96,1.07)	0.67	0.99 (0.96,1.04)	0.80

Supplementary Table 5. Baseline characteristics of participant who only used metformin monotherapy in the past year before baseline. SMD indicates standardized mean difference. GLP-1 agonists, glucagon-like peptide-1 agonists. DPP-4 inhibitors, dipeptidyl peptidase 4 inhibitors. HFRS, hospital frailty risk score. ACB score, anticholinergic burden score. SGLT2 inhibitors, sodium-glucose cotransporter-2 (SGLT2) inhibitors. The P-value for the difference among three groups were estimated by Chi-square for categorical factors and by ANOVA for continuous factors.

				Crude standardized mean difference			Weighted standardized mean difference		
	**GLP-1 agonists**	**DPP-4 inhibitors**	**Sulfonylureas**	**GLP-1 agonists vs. sulfonylureas**	**DPP-4 inhibitors vs. sulfonylureas**	**GLP-1 agonists vs. DPP-4 inhibitors**	**GLP-1 agonists vs. sulfonylureas**	**DPP-4 inhibitors vs. sulfonylureas**	**GLP-1 agonists vs. DPP-4 inhibitors**
n	3532	26581	23475						
Sex = Woman (%)	1516 (42.9)	11782 (44.3)	10560 (45.0)	0.0416	0.0133	0.0283	0.0172	0.0162	0.0009
Age at baseline (mean (SD))	71.16 (4.63)	74.24 (6.37)	73.59 (6.16)	0.4460	0.1040	0.5538	0.0249	0.0466	0.0226
Household income (%)				0.3223	0.1264	0.1942	0.0149	0.0165	0.0034
High	1564 (44.3)	9270 (34.9)	6849 (29.2)						
Low	921 (26.1)	8457 (31.8)	8315 (35.4)						
Median	1047 (29.6)	8854 (33.3)	8311 (35.4)						
Education (%)				0.3419	0.1048	0.2370	0.0009	0.0198	0.0198
Primary education	1011 (28.6)	10490 (39.5)	10347 (44.1)						
Secondary education	1651 (46.7)	11055 (41.6)	9390 (40.0)						
Tertiary education or higher	870 (24.6)	5036 (18.9)	3738 (15.9)						
Civil status = Having a partner (%)	1176 (33.3)	6793 (25.6)	4635 (19.7)	0.3107	0.1392	0.1705	0.0028	0.0017	0.0011
Enrollment year (%)				1.2655	1.0864	0.2104	0.0050	0.0257	0.0281
2010-2013	485 (13.7)	3837 (14.4)	11927 (50.8)						
2014-2017	1116 (31.6)	11963 (45.0)	9519 (40.5)						
2018-2020	1931 (54.7)	10781 (40.6)	2029 (8.6)						
Cancers = Yes (%)	333 (9.4)	2746 (10.3)	2203 (9.4)	0.0015	0.0317	0.0303	0.0093	0.0073	0.0020
Cerebrovascular diseases = Yes (%)	196 (5.5)	1886 (7.1)	1598 (6.8)	0.0523	0.0113	0.0636	0.0257	0.0324	0.0067
Hyperlipidemia = Yes (%)	473 (13.4)	3142 (11.8)	2599 (11.1)	0.0709	0.0235	0.0474	0.0122	0.0096	0.0026
Hypertension = Yes (%)	1553 (44.0)	10621 (40.0)	8580 (36.5)	0.1517	0.0702	0.0814	0.0060	0.0177	0.0116
Heart diseases = Yes (%)	975 (27.6)	6736 (25.3)	5516 (23.5)	0.0943	0.0429	0.0513	0.0044	0.0145	0.0100
Neurological disorders = Yes (%)	95 (2.7)	851 (3.2)	601 (2.6)	0.0081	0.0384	0.0303	0.0310	0.0177	0.0134
Hearing loss = Yes (%)	142 (4.0)	1223 (4.6)	1157 (4.9)	0.0439	0.0154	0.0286	0.0152	0.0200	0.0048
Mental disorders = Yes (%)	235 (6.7)	1582 (6.0)	1218 (5.2)	0.0621	0.0333	0.0289	0.0022	0.0038	0.0060
Obesity = Yes (%)	436 (12.3)	1000 (3.8)	721 (3.1)	0.3530	0.0380	0.3194	0.0033	0.0159	0.0127
Sleep disorders = Yes (%)	284 (8.0)	1018 (3.8)	729 (3.1)	0.2164	0.0396	0.1789	0.0108	0.0039	0.0147
Lifestyle-related conditions = Yes (%)	124 (3.5)	744 (2.8)	531 (2.3)	0.0746	0.0342	0.0407	0.0039	0.0009	0.0031
Antihypertensive drugs = Yes (%)	3215 (91.0)	23217 (87.3)	20081 (85.5)	0.1711	0.0527	0.1187	0.0054	0.0101	0.0047
Lipid lowering drugs = Yes (%)	2500 (70.8)	18231 (68.6)	15024 (64.0)	0.1450	0.0971	0.0478	0.0071	0.0144	0.0073
Cardiac therapy = Yes (%)	493 (14.0)	3738 (14.1)	3617 (15.4)	0.0410	0.0380	0.0030	0.0010	0.0117	0.0107
Psychotropic medications = Yes (%)	590 (16.7)	3816 (14.4)	3076 (13.1)	0.1012	0.0364	0.0649	0.0002	0.0081	0.0079

Neuropharmaceuticals = Yes (%)	265 (7.5)	1608 (6.0)	1177 (5.0)	0.1029	0.0453	0.0579	0.0152	0.0046	0.0106
Antithrombotics = Yes (%)	1754 (49.7)	13278 (50.0)	11783 (50.2)	0.0107	0.0048	0.0059	0.0054	0.0176	0.0122
Home cares = Yes (%)	248 (7.0)	3490 (13.1)	2557 (10.9)	0.1359	0.0689	0.2040	0.0088	0.0104	0.0017
Chronic morbidity index (mean (SD))	3.68 (3.02)	3.38 (2.94)	3.13 (2.77)	0.1885	0.0873	0.0998	0.0041	0.0188	0.0149
HFRS (mean (SD))	1.51 (2.63)	1.50 (2.64)	1.30 (2.35)	0.0823	0.0795	0.0025	0.0160	0.0255	0.0091
ACB score (mean (SD))	4.93 (10.50)	5.48 (14.49)	5.41 (13.83)	0.0390	0.0048	0.0432	0.0140	0.0202	0.0074
No. of diabetic complications (mean (SD))	0.13 (0.40)	0.12 (0.39)	0.12 (0.37)	0.0227	0.0073	0.0151	0.0011	0.0015	0.0004

Supplementary Table 6. Baseline characteristics of participant who ever used insulin in the past year before baseline. SMD indicates standardized mean difference. GLP-1 agonists, glucagon-like peptide-1 agonists. DPP-4 inhibitors, dipeptidyl peptidase 4 inhibitors. HFRS, hospital frailty risk score. ACB score, anticholinergic burden score. SGLT2 inhibitors, sodium-glucose cotransporter-2 (SGLT2) inhibitors. The comparison was only made between GLP-1 agonists and DPP-4 inhibitors since sulfonylureas are rarely used in combination with insulin. The P-value for the difference among three groups were estimated by Chi-square for categorical factors and by ANOVA for continuous factors.

	GLP-1 agonists	DPP-4 inhibitors	Crude standardized mean difference	Weighted standardized mean difference
			GLP-1 agonists vs. DPP-4 inhibitors	GLP-1 agonists vs. DPP-4 inhibitors
n	7087	9253	0.0892	<0.001
Sex = Woman (%)	2904 (41.0)	4200 (45.4)	0.5479	<0.001
Age at baseline (mean (SD))	71.89 (4.95)	75.11 (6.69)	0.2430	<0.001
Household income (%)				
High	2823 (39.8)	2692 (29.1)		
Low	1956 (27.6)	3341 (36.1)		
Median	2308 (32.6)	3220 (34.8)		
Education (%)			0.1909	<0.001
Primary education	2494 (35.2)	4095 (44.3)		
Secondary education	3277 (46.2)	3807 (41.1)		
Tertiary education or higher	1316 (18.6)	1351 (14.6)		
Civil status = Having a partner (%)	2221 (31.3)	1884 (20.4)	0.2527	<0.001
Enrollment year (%)			0.1499	<0.001
2010-2013	1194 (16.8)	1594 (17.2)		
2014-2017	2614 (36.9)	4315 (46.6)		
2018-2020	3279 (46.3)	3344 (36.1)		
Cancers = Yes (%)	671 (9.5)	1027 (11.1)	0.0537	<0.001
Cerebrovascular diseases = Yes (%)	582 (8.2)	989 (10.7)	0.0847	<0.001
Hyperlipidemia = Yes (%)	1572 (22.2)	1712 (18.5)	0.0915	<0.001
Hypertension = Yes (%)	3872 (54.6)	4976 (53.8)	0.0172	<0.001
Heart diseases = Yes (%)	2440 (34.4)	3315 (35.8)	0.0293	<0.001
Neurological disorders = Yes (%)	198 (2.8)	337 (3.6)	0.0481	<0.001
Hearing loss = Yes (%)	374 (5.3)	423 (4.6)	0.0326	<0.001
Mental disorders = Yes (%)	531 (7.5)	708 (7.7)	0.0060	<0.001
Obesity = Yes (%)	1034 (14.6)	650 (7.0)	0.2455	<0.001
Sleep disorders = Yes (%)	645 (9.1)	492 (5.3)	0.1467	<0.001
Lifestyle-related conditions = Yes (%)	248 (3.5)	322 (3.5)	0.0011	<0.001
Antihypertensive drugs = Yes (%)	6612 (93.3)	8573 (92.7)	0.0253	<0.001
Lipid lowering drugs = Yes (%)	5635 (79.5)	6898 (74.5)	0.1182	<0.001
Cardiac therapy = Yes (%)	1367 (19.3)	1834 (19.8)	0.0134	<0.001
Psychotropic medications = Yes (%)	1275 (18.0)	1720 (18.6)	0.0155	<0.001

Neuropharmaceuticals = Yes (%)	616 (8.7)	770 (8.3)	0.0133	<0.001
Antithrombotics = Yes (%)	4522 (63.8)	6080 (65.7)	0.0398	<0.001
Home cares = Yes (%)	919 (13.0)	2274 (24.6)	0.3006	<0.001
No. of comorbidities (mean (SD))	4.82 (3.36)	4.83 (3.35)	0.0025	<0.001
HFRS (mean (SD))	2.01 (3.20)	2.45 (3.64)	0.1291	<0.001
ACB score (mean (SD))	6.95 (16.04)	8.75 (19.20)	0.1015	<0.001
Chronic morbidity index (mean (SD))	0.60 (0.92)	0.54 (0.84)	0.0753	<0.001
Metformin = Yes (%)	4900 (69.1)	6018 (65.0)	0.0874	<0.001
SGLT2 inhibitors = Yes (%)	855 (12.1)	476 (5.1)	0.2487	<0.001
Thiazolidinediones = Yes (%)	80 (1.1)	73 (0.8)	0.0349	<0.001
Combinations of oral antidiabetic drugs = Yes (%)	211 (3.0)	174 (1.9)	0.0713	<0.001
Other antidiabetic drugs = Yes (%)	322 (4.5)	425 (4.6)	0.0024	<0.001

Supplementary Table 7. Weighted risk difference per 1,000 for dementia within 5 years from baseline between DPP-4. GLP-1, and sulfonylurea groups. GLP-1 agonists refer to glucagon-like peptide-1 agonists. DPP-4 inhibitors are dipeptidyl peptidase 4 inhibitors. The intention-to-treatment analysis included all participants, considering a five-year look-back period for eligibility assessment, while the per-protocol analysis further considered adherence to the assigned treatment during follow-up. All risk differences were estimated after weighting by the propensity scores. The analysis of metformin users only included participants who exclusively received dispensation of metformin in the year preceding baseline, while the analysis of insulin users included participants who used insulin within the year before baseline.

**Analysis**	**GLP-1 vs. sulfonylureas**	**DPP-4 vs. sulfonylureas**	**GLP-1 vs. DPP-4**
**Intention-to-treatment**			
Overall participants	-9.79 (-13.46,-6.11)	-2.86 (-5.56,-0.16)	-6.92 (-9.97,-3.87)
Metformin users	-4.99 (-9.20,-0.78)	-0.61 (-3.03,1.81)	-4.37 (-8.38,-0.36)
Insulin users	Not applicable	Not applicable	-8.41 (-12.57,-4.25)
Per-protocol			
**Overall participants**	-11.58 (-13.19,-9.97)	1.04 (-0.43,2.5)	-12.62 (-14.08,-11.16)
Metformin users	-9.51 (-12.06,-6.96)	0.65 (-1.03,2.33)	-10.16 (-12.73,-7.58)
Insulin users	Not applicable	Not applicable	-13.81 (-16.39,-11.24)

Supplementary Figure 1. Standardized mean differences for baseline characteristics before and after weighing by propensity scores.

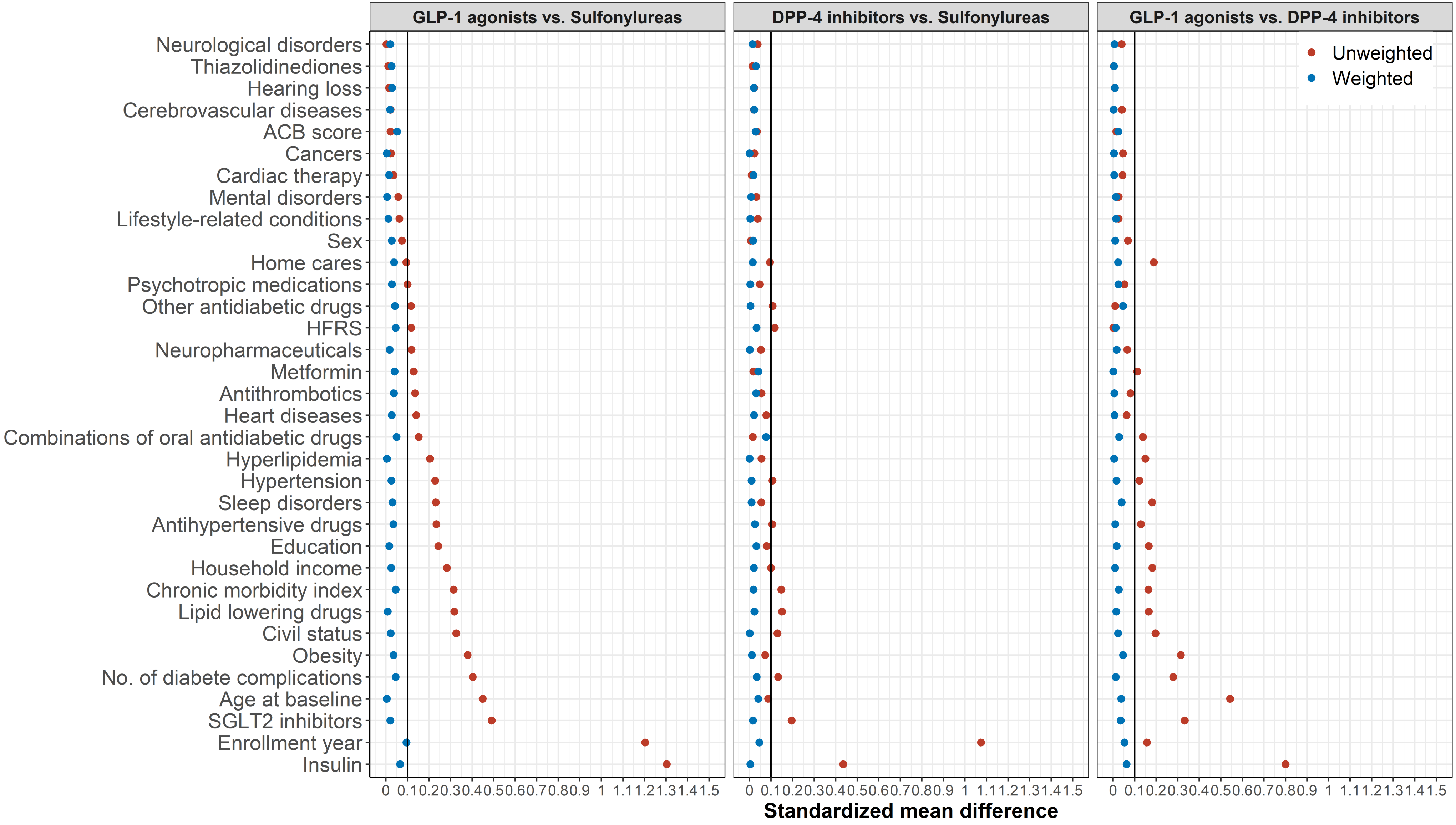

Supplementary Figure 2. Standardized mean differences for baseline characteristics before and after weighing by propensity scores for the participant who only used metformin to control their blood glucose level in the past year before baseline.

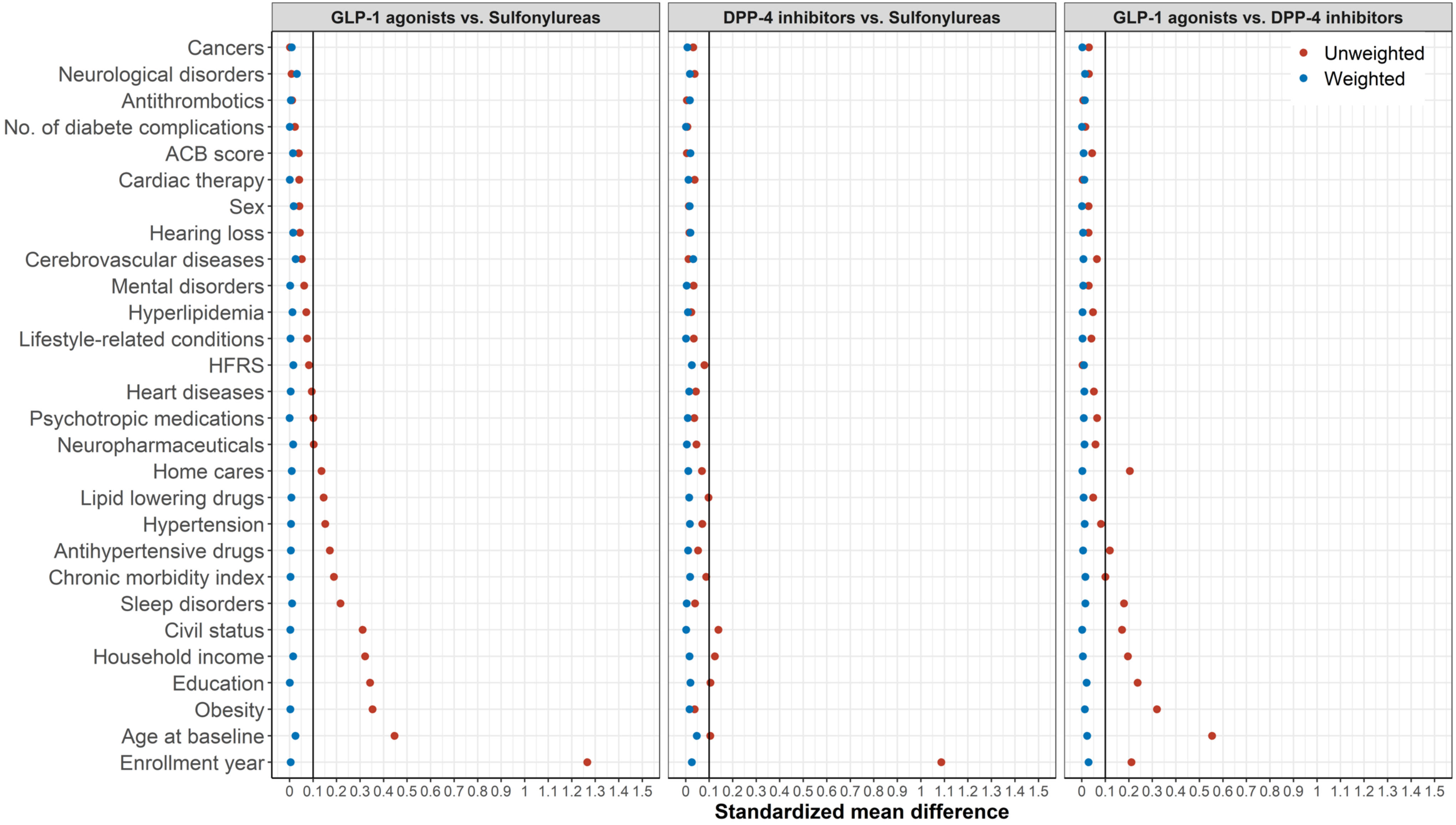

Supplementary Figure 3. Standardized mean differences for baseline characteristics before and after weighing by propensity scores for the participant who ever used insulin in the past year before baseline.

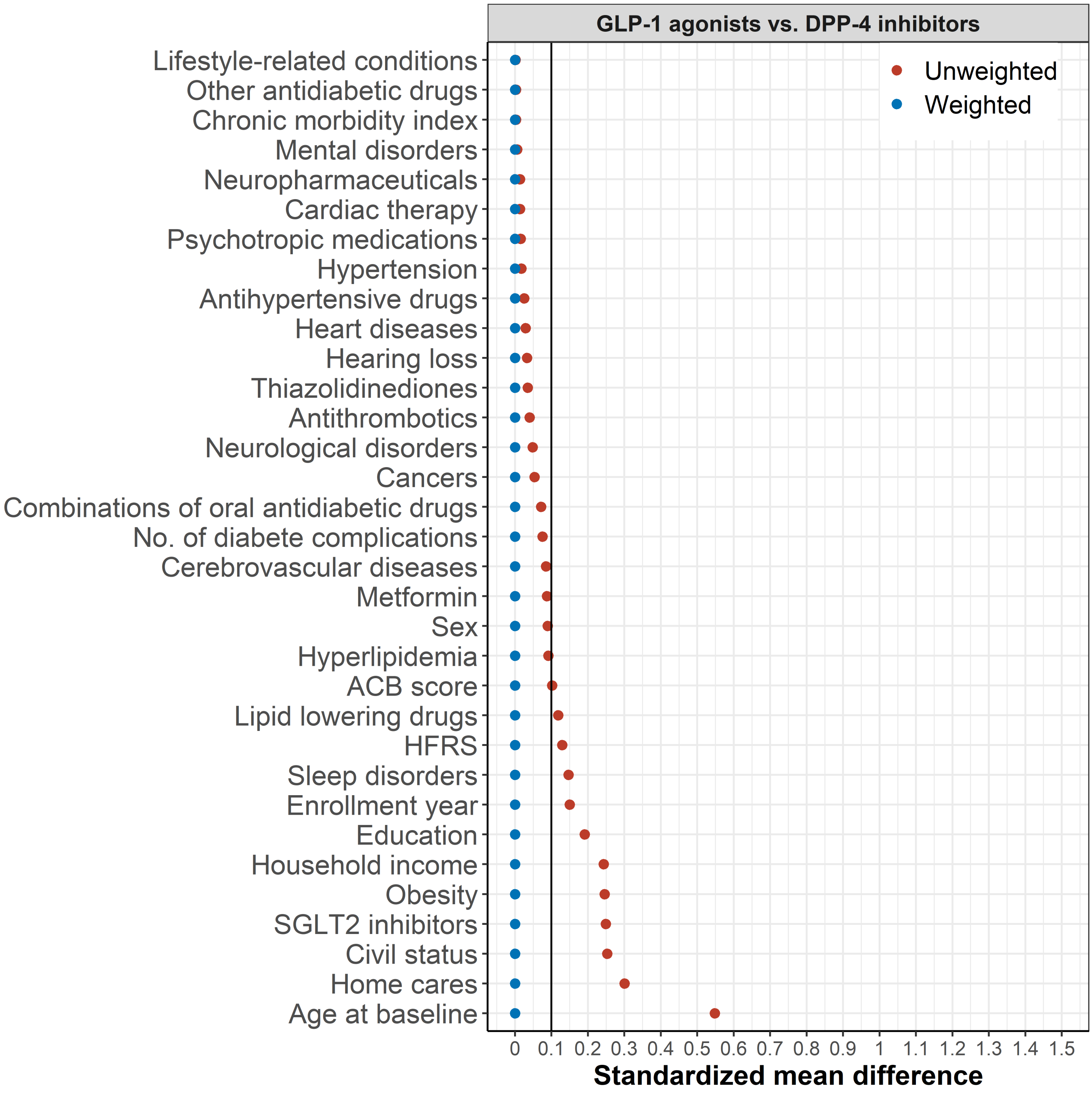

Supplementary Method

We first pooled the participants from all sequentially emulated trials. Descriptive statistics were performed to summarize the baseline characteristics of the participants. Mean and standard deviation (SD) were used for continuous variables, while frequency and proportion were used for categorical variables. To balance the characteristics between groups, we used inverse-probability weighting (IPW) with propensity scores. For the intention-to-treat analysis, we calculated overlap propensity scores for each participant. This was done by estimating the probability of being assigned to GLP-1 agonists, DPP-4 inhibitors, or sulfonylureas at baseline using a multinomial logistic regression model with predefined covariates.(1) The overlap method, compared to other weighting methods, gives more weight to participants who have more comparable characteristics for their treatment assignment, thereby avoiding extreme weights and improving the stability of the weighted estimates.(2) We estimated the standardized mean difference (SMD) between pairs of the three groups before and after IPW to assess the balance. Since liraglutide accounted for 65% of the prescription of GLP-1 agonists in our sample, we conducted a subgroup analysis focusing solely on liraglutide initiation. To account for the potential lag time of dementia diagnosis in the NPR, which a previous study suggested with an average of 5 years,(3) we conducted sensitivity analyses censoring participants who received a dementia diagnosis within 1, 3, and 5 years from baseline. Additionally, we explored the drug effects stratified by sex and age (65-74 years old and ≥75 years old). To restrict the inclusion of participants with T2DM, we also conducted a sensitivity analysis that only included participants with a T2DM diagnosis in the NPR within the past 5 years.

For per-protocol analysis, which focuses on the effect of continuous use of the assigned treatment throughout follow-up, we considered both the probability of treatment assignment and the probability of treatment adherence. First, we estimated the exposure duration of each drug dispensation using the dispensed quantity and prescribed daily dose from the NPDR. Participants in the three treatment groups were censored after one month without refilling their assigned drug or when they started taking any of the other two drugs. Next, we calculated the stabilized propensity score by inverse weighting the time-dependent probability of adherence to the protocol.(4) The stabilized weight was estimated using the formula:

$$SW_K^Z = \prod_{k=1}^{t} \frac{\Pr(Z_k = 1 | \bar{Z}_{k-1} = 1, A, V)}{\Pr(Z_k = 1 | \bar{Z}_{k-1} = 1, A, \bar{Q}_{k-1})}$$

where K is the time point during follow-up, Z is an indicator of adherence, A is the treatment group, V is a vector of time-independent covariates (including age at baseline, sex, and enrollment year), and Q is the history of time-varying covariates.(5) To improve the efficiency of this analysis, we estimated the probability of remaining on the assigned treatment for each time point using a pooled logistic model, including the number of months from baseline, baseline covariates, and the most recent measurement of time-varying covariates. A participant's weight at each time is the cumulative product of the stabilized weight of assignment to the treatment and remaining on the treatment. Weights were truncated at the 0.1st and 0.9th percentile before estimating the per-protocol effect.

We conducted the analysis for pairwise comparisons among the three medications. We used the Kaplan-Meier method to estimate the weighted cumulative hazards and the Cox proportional hazard model to estimate the weighted hazard ratios (HRs) for both intention-to-treat and per-protocol effects after IPW with the propensity scores. For absolute risk, we calculated the number of events per 1,000 person-years within a five-year period from baseline and estimated the event rate difference after IPW with the propensity score. We tested the robustness of our trial emulation strategy by conducting analyses using negative and positive control outcomes. Similar to dementia, chronic lower respiratory diseases (ICD-10: J40-47), hearing loss (H90, H91), and disorders of the lens (H25-28) are highly age-related (23-25).(6-8) However, there is no biological plausibility or prior evidence suggesting a relationship with GLP-1 agonists or DPP-4 inhibitors, so they were used as negative control outcomes. Gastrointestinal reactions, including nausea and vomiting (R11), constipation (K59.0), dyspepsia (K30), heartburn (R12), and flatulence (R14), are considered adverse effects of GLP-1 agonists and were used as positive control outcomes. Since metformin is the only first-line hypoglycemic drug and is commonly

prescribed as monotherapy in the early stages of T2DM, we conducted a sensitivity analysis specifically for individuals who had exclusively used metformin in the year before the baseline to ensure comparability of T2DM severity across the three groups (referred to as "metformin only users"). Most GLP-1 agonists in Sweden until 2020 required subcutaneous injection, which doctors may consider when prescribing this medication. Therefore, an additional sensitivity analysis was performed, including individuals who had used insulin in the year prior to the baseline (referred to as "insulin ever users"). This analysis was specifically performed to compare GLP-1 with DPP-4, as the combination of sulfonylureas with insulin is not recommended. To address potential bias in estimating standard errors caused by including the same individual multiple times in the sequential trial emulation, we used bootstrapping. This involved resampling distinct individuals 1,000 times to generate two-sided confidence intervals with a significance level of $P<0.05$.

Reference for Supplementary Method

1. Spreeuwenberg MD, Bartak A, Croon MA, Hagenaars JA, Busschbach JJ, Andrea H, et al. The multiple propensity score as control for bias in the comparison of more than two treatment arms: an introduction from a case study in mental health. Med Care. 2010;48(2):166-74.
2. Mlcoch T, Hrnciarova T, Tuzil J, Zadak J, Marian M, Dolezal T. Propensity Score Weighting Using Overlap Weights: A New Method Applied to Regorafenib Clinical Data and a Cost-Effectiveness Analysis. Value in Health. 2019;22(12):1370-7.
3. Rizzuto D, Feldman AL, Karlsson IK, Dahl Aslan AK, Gatz M, Pedersen NL. Detection of Dementia Cases in Two Swedish Health Registers: A Validation Study. J Alzheimers Dis. 2018;61(4):1301-10.
4. Hernán MA, Robins JM. Per-Protocol Analyses of Pragmatic Trials. New England Journal of Medicine. 2017;377(14):1391-8.
5. Robins JM, Hernán MÁ, Brumback B. Marginal Structural Models and Causal Inference in Epidemiology. Epidemiology. 2000;11(5).
6. Rojas M, Mora AL, Kapetanaki M, Weathington N, Gladwin M, Eickelberg O. Aging and Lung Disease. Clinical Impact and Cellular and Molecular Pathways. Ann Am Thorac Soc. 2015;12(12):S222-7.
7. Petrash JM. Aging and age-related diseases of the ocular lens and vitreous body. Invest Ophthalmol Vis Sci. 2013;54(14):Orsf54-9.
8. Liu XZ, Yan D. Ageing and hearing loss. The Journal of Pathology. 2007;211(2):188-97.
