# Supplementary material for: Comparative effectiveness of glucagon-like peptide-1 agonists, dipeptidyl peptidase-4 inhibitors, and sulfonylureas on the risk of dementia in older individuals with type 2 diabetes in Sweden: an emulated trial study
Source: eClinicalMedicine. 2024 Jun 20;73:102689. doi: 10.1016/j.eclinm.2024.102689 (PMC11490655; doi:10.1016/j.eclinm.2024.102689)
Supplement: Translated abstract in Swedish [file mmc2.docx]

*The following translations in Swedish were submitted by the authors and we reproduce them as supplied. They have not been peer reviewed. Our editorial processes have only been applied to the original abstract in English, which should serve as reference for this manuscript.*

**Bakgrund**

Det är okänt om användande av glukagonliknande peptid-1 (GLP-1) agonister, dipeptidylpeptidas-4 (DPP-4) hämmare och sulfonylureor påverkar risken för demens hos äldre individer med typ 2-diabetes mellitus (T2DM).

**Metoder**

Vi simulerade en klinisk prövning med hjälp av data från Svenska nationella register från 1 januari 2010 till 30 juni 2020. Invånare som var 65 år eller äldre med T2DM och behandling med antingen GLP-1 agonister, DPP-4 hämmare eller sulfonylureor följdes upp i maximalt 10 år för att bedöma risken för demens. Personer som redan vid starten hade demens, använde de tre läkemedelsklasserna eller hade kontraindikationer exkluderades. Balans mellan grupperna uppnåddes genom att vikta olika kovariater. Alla inkluderade personer användes för intention-to-treat analys, medan per-protokoll beräknades med deltagare som följde den tilldelade behandlingen.

**Resultat**

Vi inkluderade totalt 88,381 deltagare som fick recept på GLP-1 agonister (n=12,351), DPP-4 hämmare (n=43,850) eller sulfonylureor (n=32,216) vid baslinjen och följdes upp i genomsnitt 4,3 år. Totalt utvecklades 4,607 demensfall under uppföljningen: 278 för GLP-1 agonist initierare (incidence rate: 6.7 per 1000 personår), 1,849 för DPP-4 hämmare initierare (IR: 11.8) och 2,480 för sulfonylurea initierare (IR: 13.7). I intention-to-treat analysen var initiering av GLP-1 agonister associerad med en reducerad risk för demens jämfört med sulfonylureor (Hazard ratio: 0.69, 95% CI: 0.60-0.79, p<0.0001) och DPP-4 hämmare (HR: 0.77, 95% CI: 0.68-0.88, p<0.0001), efter justering för ålder, inskrivningsår, kön, socioekonomiska faktorer, hälsotillstånd och tidigare läkemedelsanvändning. Dessa resultat var samstämmiga i ytterligare analyser inklusive per-protokoll analysen (HR för sulfonylureor: 0.41, 95% CI: 0.32-0.53, p<0.0001; HR för DPP-4 hämmare: 0.38, 95% CI: 0.30-0.49, p<0.0001).

**Tolkning**

Vår forskning tyder på att användande av GLP-1 agonister var associerade med en minskad risk för demens jämfört med användande av sulfonylureor eller DPP-4 hämmare hos äldre individer med T2DM. Kliniska studier behövs för att validera dessa fynd.

**Finansiering**

Vetenskapsrådet, Karolinska Institutet, National Institute on Aging, National Institutes of Health, och Riksbankens Jubileumsfond.

**Nyckelord**

läkemedel mot diabetes, demens, simulerad klinisk prövning
